# Poor risk factor control in outpatients with diabetes mellitus type 2 in Germany: The DIAbetes COhoRtE (DIACORE) study

**DOI:** 10.1371/journal.pone.0213157

**Published:** 2019-03-21

**Authors:** Myriam Rheinberger, Bettina Jung, Thomas Segiet, Johann Nusser, Günther Kreisel, Axel Andreae, Jochen Manz, Gerhard Haas, Bernhard Banas, Klaus Stark, Alexander Lammert, Mathias Gorski, Iris M. Heid, Bernhard K. Krämer, Carsten A. Böger

**Affiliations:** 1 Department of Nephrology, University Hospital Regensburg, Regensburg, Germany; 2 Diabetologische Schwerpunktpraxis Dres. Segiet, Gleixner und Bode, Speyer, Germany; 3 Diabetes Zentrum Regensburg, Regensburg, Germany; 4 Praxis Dr. med. Axel Andrae, Regensburg, Germany; 5 Praxis Dr. med. Jochen Manz, Regensburg, Germany; 6 Gemeinschaftspraxis Dr. med. Gerhard Haas, Sabine Haas, Regensburg, Germany; 7 Department of Genetic Epidemiology, University of Regensburg, Regensburg, Germany; 8 Vth Department of Medicine, Medical Faculty Mannheim of the University of Heidelberg, Mannheim, Germany; 9 Department of Nephrology, Diabetology and Rheumatology, Klinikum Traunstein, Kliniken Südostbayern, Traunstein, Germany; 10 KfH Kidney Center Traunstein, Traunstein, Germany; Baker IDI Heart and Diabetes Institute, AUSTRALIA

## Abstract

**Introduction:**

Patients with diabetes mellitus type 2 (DM2) are at high risk for micro- and macrovascular disease. Here, we explore the degree of traditional risk factor control in the baseline visit of a cohort of DM2 outpatients.

**Methods:**

DIACORE (DIAbetes COhoRtE) is a prospective cohort study of 3000 adult DM2 outpatients. Here, we present results from the baseline visit. Sociodemographic and anthropometric variables, cardiovascular risk factors, comorbidities and medication were assessed by interview and medical exams. Serum-creatinine based estimated glomerular filtration rate (eGFRcrea) and urinary albumin-creatinine ratio (UACR) were determined for classification of chronic kidney disease (CKD). The proportion of patients with adequate control of traditional risk factors (blood pressure<140/90mmHg, HbA1c<7.5%, LDL<100mg/dl) was calculated in 2892 patients with non-missing data in 9 relevant variables within each KDIGO 2012 CKD class.

**Results:**

In the analyzed baseline data (n = 2892, 60.2% men), mean (standard deviation) values for age, DM2 duration and HbA1c were 65.3 (9.3) years, 10.3 (8.4) years and 6.9% (1.1) respectively. Of these 2892 patients, 18.7% had CKD stage 3 or higher, 25.7% had UACR≥30mg/g. Adequate blood pressure, HbA1c and LDL control was achieved in 55.7%, 78.5% and 34.4%, respectively. In 16.4% of patients (473), all three risk factors were below recommended targets. The proportion of adequate risk factor control was similar across KDIGO eGFRcrea classes. Adequate blood pressure and HbA1c control were significantly associated with lower UACR category without and with controlling for other risk factors (p<0.0001, p = 0.0002, respectively).

**Conclusion:**

In our study of patients with diabetes mellitus type 2, we observed a low level of risk factor control indicating potential for risk reduction.

## Introduction

Prevalence of diabetes mellitus type 2 (DM2) is increasing worldwide and is already a major global public health issue [1, 2]. Patients with DM2 have an increased risk for micro- and macrovascular diseases such as coronary heart disease, peripheral arterial disease, retinopathy, chronic kidney disease (CKD) and end-stage renal disease (ESRD). Indeed, in every third patient with renal replacement therapy, diabetic nephropathy is claimed to be the underlying cause, making it the single most frequent cause of ESRD [3–5]. Similarly, DM2 patients have an up to four-fold risk of developing coronary heart disease and a significantly increased risk for cardiovascular mortality [6].

Micro- and macrovascular complications in DM2 patients have been linked to cardiovascular risk factors, specifically the degree of hypertension, glucose control, dyslipidemia and tobacco consumption. Pharmaceutical and behavioral management strategies for optimal control of these factors have been developed in interventional studies, leading to a reduction of micro- and macrovascular end points [7–20]. However, significant risk for these end points remains, suggesting other components such as limited adherence to therapies and thus risk factor control.

Cohort studies of the general population such as the KORA, Framingham Heart and SHIP studies are well-suited to generate hypotheses on disease mechanisms in general populations, owing to the combination of high sample size, standardized protocols and thus comparability with other cohort studies. However, the number of DM2 patients in these cohorts is usually limited since less than 10% of the general population is affected by DM2. Accordingly, prevalence estimates of cardiovascular risk factors and their complications in the DM2 subpopulation may not be reliable.

We thus established the DIAbetes COhoRtE study (DIACORE) [21], a cohort study of 3000 patients with DM2. The recruitment strategy was designed to obtain a cohort of outpatients with DM2 in Germany that is exposed to diabetes care in real-life conditions. In order to determine whether risk factor control bears the potential for improving care of patients with diabetes, we analyzed the degree of control of pharmacologically modifiable traditional risk factors (blood pressure, HbA1c, LDL) in the cross-sectional baseline data of DIACORE overall and by CKD subgroups.

## Methods

### Study design

The DIAbetes CohoRtE (DIACORE) is a two-center observational cohort study of outpatients with prevalent diabetes mellitus type 2 (DM2) with planned long term prospective follow-up of at least 10 years. Its design and protocol have been described in detail previously [21]. The study and its protocol have been approved by the participating Universities’ Ethics Committees and is in accordance with the Declaration of Helsinki. The study is registered at the German Registry of Clinical Trials (DRKS00010498) and at the International Clinical Trials Registry Platform of the World Health Organisation. Here, we present data from the baseline visit of DIACORE.

### Inclusion and exclusion criteria

Inclusion criteria at baseline were age ≥ 18 years, prevalent DM2 and Caucasian descent. DM2 was defined as the use of blood glucose lowering medication, fasting glucose values ≥ 126mg/dl,a 2-hour glucose measure ≥ 200mg/dl in the oral glucose tolerance test or HbA1c ≥ 6,5% (≥ 48mmol/mol). Exclusion criteria at baseline were chronic renal replacement therapy, active malignancy in the last 5 years (2 years for basalioma or prostate carcinoma), autoimmune disease with possible kidney involvement, haemochromatosis, known pancreatoprivic diabetes, type 1 diabetes mellitus, acute infection, HIV or chronic virus hepatitis.

### Study examinations

At each visit including the baseline visit reported here, a core phenotyping protocol is performed that includes a standardized interview using an electronic case report form (eCRF; MEDEORA GmbH, Köln, Germany), a physical examination, the determination of standard laboratory parameters from serum, urine and whole blood in a central laboratory and biobanking of biomaterials.

In the interview, information is obtained on sociodemographic and lifestyle factors, cardiovascular risk factors and cardiovascular as well as renal comorbidities. Further, information on prescribed and over-the-counter medication is obtained.

In the physical examination, we obtained measurements of blood pressure, pulse, body weight and height, and waist and hip circumference, using methods and instruments as described previously [21]. The mean of the second and third of three blood pressure measurements obtained after a five minute rest is defined as the mean blood pressure value for each patient. Hypertension was defined as the use of at least one blood pressure lowering medication or the presence of mean systolic ≥140 mmHg or diastolic blood pressure ≥90 mmHg. Samples of spot urine, lithium-heparin-anticoagulated serum, EDTA-anticoagulated whole blood and sodium fluoride anticoagulated whole blood were shipped by overnight express at 4°C to a central laboratory for measurement of a standard laboratory panel, as described previously [21]. One serum sample per patient was frozen at -20°C and shipped on dry ice for measurement of serum insulin. **S1 Table** provides details on the assays and analyzers used for each parameter determined. Additional samples of serum, spot urine and plasma were aliquoted and stored at -80°C for future use in research projects. Further, EDTA-anticoagulated whole blood was drawn and stored at -20°C for future DNA isolation.

We used serum creatinine measured with an IDMS traceable assay (Creaplus Tina-quant, Roche) to estimate glomerular filtration rate (eGFRcrea) using the CKD-EPI 2009 formula [22]. Serum cystatin C measured with the Cystatin C Tina Quant assay was used to estimate glomerular filtration rate (GFR) with the CKD-EPI 2012 formula (eGFRcys) [23]. Urinary albumin creatinine ratio (UACR) was determined from urinary creatinine and albumin measurements from spot midstream urine. The lower limit of detection of the assay for albumin in urine was 3 mg/l. Following common practice in the CKD Prognosis Consortium, values were set to 3mg/L in individuals with urine albumin <3mg/L [24].

Patients were classified into five eGFRcrea and three UACR categories according to the KDIGO 2012 definition [25]. For descriptive statistics, we subdivided the G3 eGFRcrea category into category G3a (eGFRcrea 45–59 ml/min/1.73m^2^) and G3b (eGFRcrea 30–44 ml/min/1.73m^2^). We defined chronic kidney disease (CKD) as eGFRcrea < 60 ml/min/1.73m^2^ [25] and diabetes-associated kidney disease (DKD) as UACR ≥ 30mg/g, irrespective of eGFRcrea [25]. Adequate control of pharmacologically modifiable vascular risk factors was defined as LDL < 100mg/dL, HbA1c < 7.5%, or systolic blood pressure < 140 mmHg and diastolic blood pressure < 90mmHg [26, 27, 28].

Cardiovascular and renal events were validated by obtaining the corresponding physician report where possible. Thus, validated cardiovascular and renal events are a subset of all patient-reported events. In this manuscript, we report only medically validated events. “Macrovascular comorbidity” is defined as the composite of myocardial infarction, myocardial intervention (percutaneous coronary angioplasty with or without stent application), coronary bypass-operation, peripheral arterial intervention or bypass-operation, amputation, stroke or intervention or operation of the carotid artery. “Microvascular comorbidity” is defined as the composite of the presence of DKD or retinal laser therapy for diabetic retinopathy.

### Statistical analysis

We did not impute missing data in variables. To obtain consistency in numbers across analyses, we limited the analysis of control of cardiovascular risk factors to individuals with non-missing data in a set of 9 key variables (age, gender, diabetes duration, blood pressure, body mass index, HbA1c, LDL, eGFRcrea and UACR).

Descriptive statistics of continuous variables are expressed as mean ± standard deviation for normally distributed variables or median [25% interquartile range] for variables with non-normal distribution, for categorical variables as relative frequencies (%). Comparisons between groups were performed with a t-test or by ANOVA for continuous variables and with Pearson’s χ^2^ test for categorical variables. Statistical significance was defined as p<0.05.

For risk factors showing an increasing or decreasing trend of adequate control across UACR or eGFRcrea categories, we tested the proportion of adequate risk factor control by KDIGO UACR or eGFRcea category (UACR: <30mg/g = 0; 30-300mg/g = 1; >300mg/g = 2; eGFRcrea: ≥90 ml/min/1.73m^2^ = 1; 60–89 ml/min/1.73m^2^ = 2, 30–59 ml/min/1.73m^2^ = 3; 15–29 ml/min/1.73m^2^ = 4; <15 ml/min/1.73m^2^ = 5). For this, we used ordinal logistic regression with three models of adjustments: (i) a univariable model without any adjustment, (ii) a model 1 adjusting for age, sex, and diabetes duration, and (iii) a model 2 additionally adjusting for eGFRcrea (or lnUACR), BMI, waist-hip-ratio, smoking status (ever smoker = 1; never smoker = 2), and HbA1c (for the analysis of adequacy of blood pressure and lipid control) or systolic and diastolic blood pressure (for the analysis of HbA1c or lipid control) or LDL (for the analysis of adequacy of blood pressure and HbA1c control).

All computations were performed in JMP Version 8.0.2 (SAS Institute).

## Results

### DIACORE study sample overview

Patient recruitment at the baseline visit (Visit 1) was performed from 2/2010 through 8/2014 in two University based study centers in Germany through direct written or spoken invitation to participate, press articles, flyers or public presentations (**S1 Fig**). For recruiting in the Regensburg region, 22,932 written invitations were mailed, mostly flanked by press releases in local media: 16,882 written invitations were sent by mail by 5 medical insurance companies, 1,700 invitations were sent by 2 diabetologists, 400 invitations were sent by the Bavarian Diabetes Patient Group (“Diabetikerbund Bayern”) and 3,950 invitations were sent to patients who had previously received inpatient treatment at the University Hospital Regensburg’s Internal Medicine Departments 1 and 2 (Cardiology, Endocrinology, Gastroenterology, Hepatology, Nephrology, Pulmonology). Further, 10 general practitioners advertised the DIACORE study by presenting study flyers in their waiting room and by actively inviting the patients to participate. For recruiting in the Mannheim region, 2,000 patients with DM2 were invited to enroll by a DIACORE study nurse in a diabetologist doctor’s office in the city of Speyer (Dres. Segiet, Gleixner and Bode). Overall, 4226 patients contacted DIACORE (including those directly asked in the Speyer diabetology office), of which 1226 did not fulfill the inclusion criteria or had at least one exclusion criterion. Thus, a total of 3000 patients were included in the DIACORE study. **S2 Table** provides details of how patients were recruited into DIACORE.

The use of diabetologists, urologists and nephrologists by patients in DIACORE differed by discipline: overall, 1586 (52.9%) patients reported being in regular care of a diabetologist. While 57.9% (1737) of participants had previously consulted a urologist (n = 1171, 65.0% of all men; n = 566, 47.2% of all women), only 13% (389) had ever consulted a nephrologist (n = 215, 11.9% of all men, n = 174, 14.5% of all women). In patients with CKD this proportion was higher: 16.2% (n = 100) and 26.2% (n = 34) of patients with micro- and macroalbuminuria respectively and 23.8% (n = 135) with eGFR <60 ml/min/1.72m^2^ had consulted a nephrologist.

For the following cross-sectional analyses of cardiovascular risk factors and risk factor control by medication in DM patients and its link to kidney function, we restricted the analyzed sample to the 2892 DIACORE participants with data available for age, gender, diabetes duration, BMI, blood pressure, LDL, HbA1c, eGFRcrea and UACR at the baseline visit. The analyzed sample included 1740 (60%) men, with mean age (±standard deviation, SD) of 65.3±9.3 years, mean diabetes duration 10.3±8.4 years (median 8.3, 25% IQR 4.1–14.5; range 0 to 57.7 years) and mean HbA1c of 6.9%±1.1 (median 6.6%, 25% IQR 6.2%-7.4%, range 4.2%-16.7%; **Table 1**). The majority of patients were in disease management programs (76.9%). A full description of baseline values of variables and distributions for all 3000 DIACORE participants is given in **S3 and S4 Tables**, showing good comparability between the full study sample and the 2892 patients analysed here.

**Table 1:** Clinical characteristics and cardiovascular risk factors of the analysed 2892 DIACORE participants.

	Total
n	2892
Male	1740 (60.2%)
Age, years	65.3±9.3
Diabetes duration, years §	8.3 (4.1–14.6)
Disease management program, n (%)	2225 (76.9%)
**Cardiovascular risk factors**	
HbA1c, %§	6.6 (6.2–7.4)
HbA1c, mmol/mol §	49 (44–57)
HbA1c < 7.5%, n (%) §	2269 (78.5%)
Glucose in patients fasting >12 h*, mg/dl §	122 (103.1–147.7)
Glucose in nonfasting patients$, mg/dl §	115 (94.8–154.3)
Systolic blood pressure / diastolic blood pressure, mmHg	139.0±18.2 / 76.5±10.5
Blood pressure <140/90 mmHg, n (%)	1610 (55.7%)
BMI, kg/m^2^	31.4±5.7
WHR	0.96±0.09
HDL, mg/dl	52.9±15.3
LDL, mg/dl	117.9±36.9
LDL < 100 mg/dl, n (%)	995 (34.4%)
Never-smokers, n (%)	1233 (42.6%)
Current smokers, n (%)	358 (12.4%)
Former smokers, n (%)	1301 (45.0%)

Values provided are mean±SD if not stated otherwise.

§ median (IQR).

* 1608 patients (55.6%) were fasting > 12h. $ 940 (32.5%) were nonfasting.

A total of 108 (3.6%) of the full cohort sample of 3000 DIACORE patients had missing data for at least one of the following variables: BMI (n = 15), LDL (n = 7), blood pressure (n = 2), eGFR (n = 7), HbA1c (n = 8) or UACR (n = 87). None had missing data for age, sex or diabetes duration.

### Cardiovascular risk factors and comorbidities

A high proportion of the 2892 analysed DIACORE participants exhibited cardiovascular risk factors. 1575 (54.5%) were obese, 2539 (87.8%) patients had hypertension, 1659 (57.4%) were current or former smokers, 623 (21.5%) had HbA1c>7.5%, and LDL was above 100mg/dl in 1897 (65.6%). Further detail on cardiovascular risk factors for the analyzed DIACORE participants is provided in **Table 1,** showing comparable data to the full DIACORE study of 3000 patients **(S3 Table).**

**Fig 1:**
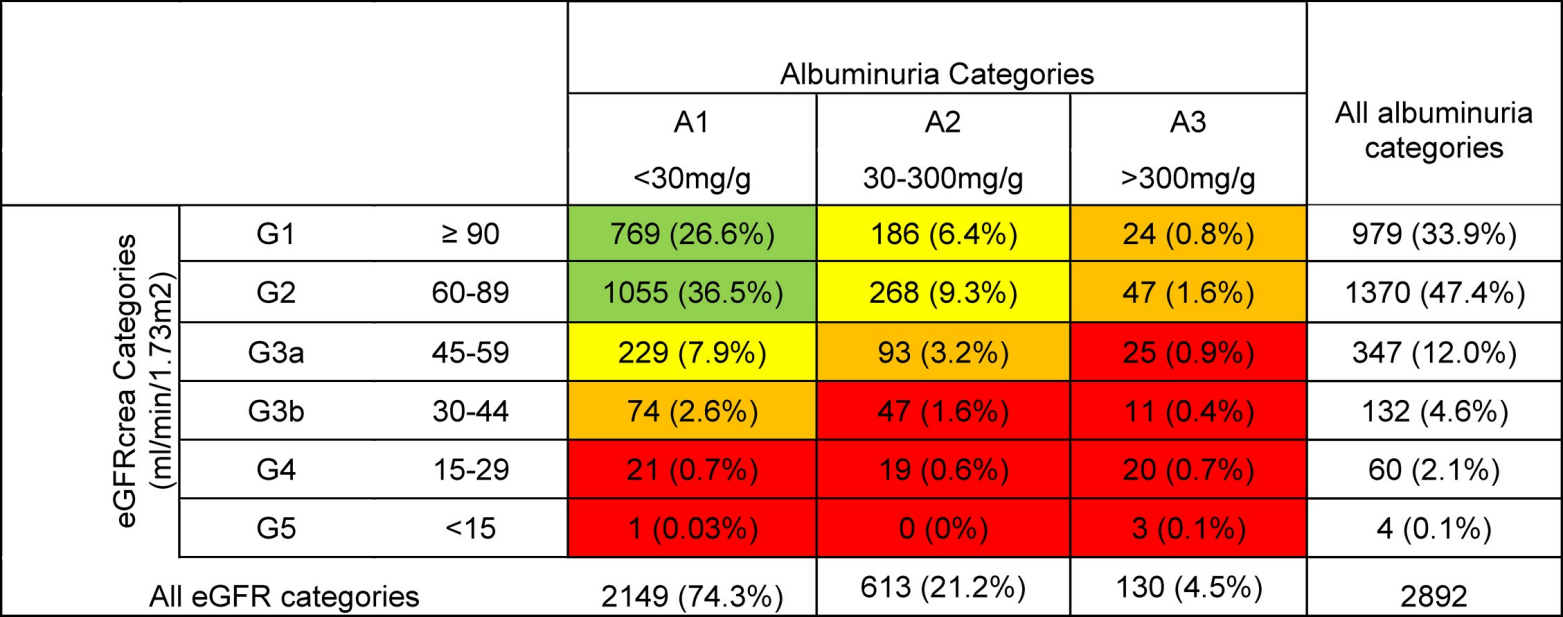
Distribution of 2892 analyzed patients according to eGFRcrea and albuminuria categories according the KDIGO 2012 CKD classification [25]. Field coloring indicates risk for progression of CKD according to the 2012 KDIGO guideline (green: low risk, yellow: moderately increased risk, orange: high risk, red: very high risk) [25].

We also observed a substantial degree of micro- and macrovascular comorbidities (**Table 2).** While only 109 (3.8%) patients reported previous laser therapy for diabetic retinopathy, DKD, defined as UACR≥30mg/g, was observed in 743 (25.7%) patients, and 1068 (36.9%) had CKD according to KDIGO 2012 **(Fig 1).** Of the patients with eGFRcrea ≥60 ml/min/1.73m^2^ 1824 (63.1%) did not have elevated UACR and thus did not fulfill the KDIGO definition criteria of CKD. The distribution of patients across the KDIGO 2012 eGFRcrea and UACR categories is shown in **Fig 1.** The gender-specific distribution of patients across KDIGO 2012 categories is shown in **S2 Fig.** Reduced kidney function (eGFRcrea<60 ml/min/1.73m^2^) was associated with DKD in a minority of patients: while a total of 543 (18.8%) had an eGFRcrea of <60ml/min/1.73m^2^, only 218 (7.5%) patients had an eGFRcrea of <60ml/min/1.73m^2^ and UACR≥30mg/g and 147 (5.1%) had a very high risk of progression of CKD according to KDIGO guidelines ([25], **Fig 1, Table 2).** The frequency of CKD in the full data set of 3000 DIACORE patients was comparable **(S4 Table).**

**Table 2:** Kidney function, micro- and macrovascular morbidity in the analysed 2892 DIACORE participants. For continuous variables, mean±SD and median (IQR) are provided.

**Kidney function and albuminuria**	
Serum creatinine, mg/dl	0.96±0.4; median 0.89 (0.75–1.06)
Serum cystatin C, mg/dl	1.07±0.4; median 0.98 (0.86–1.16)
eGFRcrea CKD-EPI, ml/min/1.73m^2^	78.6±20.3; median 82.2 (66.0–93.4)
eGFRcys CKD-EPI, ml/min/1.73m^2^	74.7±22.5; median 75.7 (59.7–91.1)
UACR, mg/g	76.1±343.3; median 10.1 (4.8–31.1)
**Microvascular comorbity**	**794 (27.5%)**
Diabetes associated kidney disease	
eGFRcrea <60 ml/min/1.73m^2^ or UACR≥30mg/g, n (%)	1068 (36.9%)
CKD stage 3 or higher (eGFRcrea<60 ml/min/1.73m^2^), n (%)	543 (18.8%)
UACR 30–300 mg/g, n (%)	613 (21.2%)
UACR>300mg/g, n (%)	130 (4.5%)
Diabetic retinopathy	
Previous retinal laser therapy, n (%)	109 (3.8%)
**Macro-vascular complications**	**718 (24.8%)**
Myocardial infarction, n (%)	249 (8.6%)
Operative myocardial revascularisation, n (%)	192 (6.6%)
Percutaneous coronary intervention, n (%)	388 (13.4%)
Stroke, n (%)	189 (6.5%)
Operative or percutaneous carotid intervention, n (%)	74 (2.6%)
Revascularization lower extremities, n (%)	48 (1.7%)
Amputation, n (%)	59 (2.0%)

A total of 108 (3.6%) of the full cohort sample of 3000 DIACORE patients had missing data for at least one of the following variables: BMI (n = 15), LDL (n = 7), blood pressure (n = 2), eGFR (n = 7), HbA1c (n = 8) or UACR (n = 87). None had missing data for age, sex or diabetes duration.

Overall, 24.8% (718) of the 2892 analyzed patients had at least one of the following: previous myocardial infarction, stroke, operative/percutaneous coronary or carotid intervention, revascularization or amputation of lower extremities. **Table 2** provides more detail for each of the macrovascular comorbidities.

### Medication

The majority of patients reported taking several prescription drugs: overall, the 2892 DIACORE patients were regularly taking a mean of 6.4±3.4 prescription drugs (median = 6; 25% IQR 4–9; range: 0–21), and only 35 (1.2%) of patients reported taking no prescription drug. In addition, a total of 1450 (50.1%) patients reported taking at least one “on demand” medication (mean 0.73, median = 0; 25%; IQR 0–1; range 0–7).

We further analysed treatment modalities for diabetes, hypertension and hyperlipoproteinemia to assess patterns potentially affecting degree of risk factor control.

A total of 2537 (87.7%) of patients reported using glucose lowering medication. The spectrum of diabetes treatment modalities was diverse (Table 3), with 1540 (53.3%) using only oral antidiabetic medication, 365 (12.6%) only insulin, 61 (2.1%) other injectable antidiabetic drugs (GLP-1 receptor agonists) and 577 (20.0%) taking combinations of oral and injectable drugs. Among the 2162 patients under oral antidiabetic medication, most reported using a biguanide (n = 1862, 86.1%). **Table 3** provides further details on the use of different oral glucose lowering medication.

**Table 3:** Medication in the 2892 analysed DIACORE patients.

	Total
**Glucose lowering medication n, (%)**	2537 (87.7%)
Injectable, n (%)	993 (34.3%)
Insulin, n (%)	942 (32.6%)
GLP-1 receptor agonists, n (%)	61 (2.1%)
Oral Antidiabetic medication, n (%)	2162 (74.8%)
Biguanides, n (%)	1862 (64.4%)
Sulfonylureas, n (%)	548 (18.9%)
DPP4-Inhibitor, n (%)	560 (19.4%)
Glinides, n (%)	121 (4.0%)
Alphaglukosidase-Inhibitors, n (%)	50 (1.7%)
SGLT-2-Inhibitors, n (%)	11 (0.4%)
**Antihypertensive medication, n (%)**	2339 (80.8%)
RAAS-Inhibitor, n (%)	1942 (67.2%)
ACE-Inhibitor, n (%)	1236 (42.7%)
Angiotensin receptor blocker, n (%)	730 (25.2%)
Renin Inhibitor, n (%)	33 (1.1%)
Calcium channel blocker, n (%)	807 (27.9%)
Beta-Blocker, n (%)	1389 (48.0%)
Diuretics, n (%)	1206 (41.7%)
**Lipid-lowering medication, n (%)**	1416 (49.0%)
Statin, n (%)	1364 (47.2%)
Fibrate, n (%)	66 (2.3%)

Non-use of glucose lowering treatment was not limited to patients with HbA1c<6.5%: of 1179 (40.8%) patients with an HbA1c<6.5%, 262 (22.2%) were not on glucose lowering drugs. Of the 1090 (37.7%) patients with an HbA1c of 6.5–7.5% and the 623 (21.5%) patients with an HbA1c>7.5%, 69 (6.3%) and 24 (3.9%) did not use glucose lowering drugs respectively.

A total of 2342 (81.0%) of patients reported taking at least one antihypertensive drug, indicating a high level of at least some treatment of hypertension **(Table 3)**. Of 1282 (44.3%) patients with blood pressure levels at or above 140 mmHg systolic or 90 mmHg diastolic, 197 (15.4%) were without blood pressure lowering medication and 286 (22.3%), 338 (26.4%), 278 (21.79%) and 183 (14.3%) had 1, 2, 3 or at least four blood pressure lowering drugs respectively. In patients taking at least one blood pressure lowering medication, the mean blood pressure was 140/76 mmHg.

In contrast, only 1364 (47.2%) patients reported taking a lipid lowering medication. Of the 1914 (66.27%) patients with LDL >100mg/dl, 1254 (65.5%) were without statin. In patients taking a statin, mean LDL was 102.3 ± 31.2 mg/dl (range: 17.0–244.0 mg/dl), with 649 (47.6%) having an LDL >100mg/dl.

Medication use in the full sample of 3000 DIACORE patients was similar (**S5 Table**).

### Control of risk factors

**S6 Table** provides the distribution of cardiovascular risk factors at baseline by each of the 5 eGFR and 3 UACR categories as defined by KDIGO [25].

Overall, the control of pharmacologically modifiable risk factors (blood pressure, HbA1c, LDL) was not achieved in a large proportion of patients: in the 2892 analyzed DIACORE patients, adequate control of blood pressure, HbA1c and LDL was achieved in 1610 (55.7%), 2269 (78.5%) and 995 (34.4%) respectively. In only 473 (16.4%) patients, all three risk factors were below recommended targets. The proportions of risk factor control between the 2225 (76.9%) patients enrolled in a disease management program and those not enrolled were similar (**S7 Table).**

In the subset of 743 patients with DKD (25.7%, defined as UACR≥30mg/g), which are at particularly high risk of CKD progression and cardiovascular events, blood pressure and HbA1c targets were nominally less frequently achieved (44.0% and 70.7% respectively), while LDL goal was achieved nominally more frequently (39.4%) than in the full analysed sample of 2892 patients.

**Fig 2:**
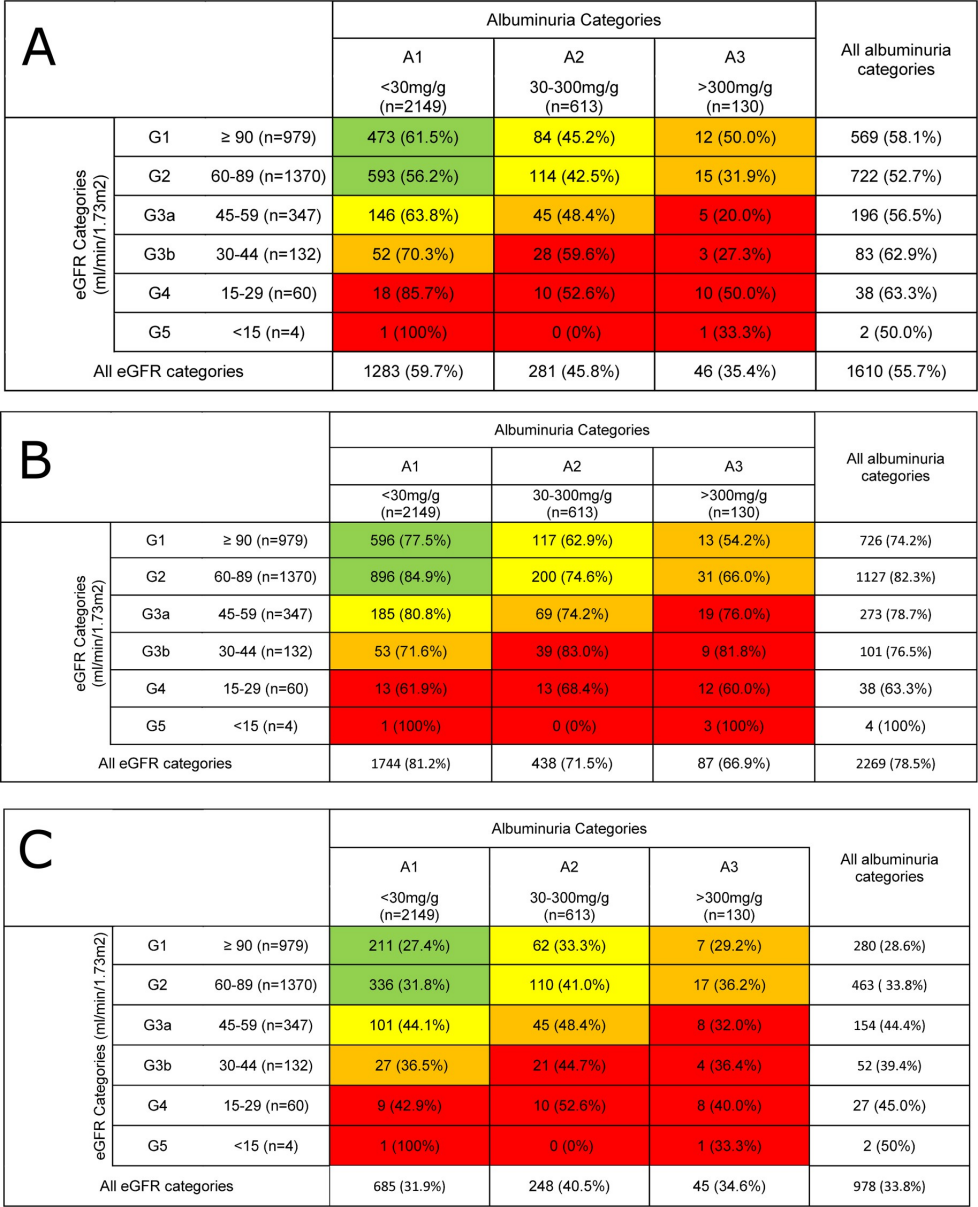
Distribution of analyzed 2892 patients within recommended targets for traditional cardiovascular risk factors by KDIGO 2012 CKD category.

The proportion of patients with adequate blood pressure control decreased across increasing (“less healthy”) KDIGO UACR categories A1-A3 (59.7%, 45.8% and 35.4%, p<0.0001, **Fig 2A**), Similarly, adequate HbA1c control (HbA1c <7.5%) decreased across increasing UACR categories (81.2%, 71.5% and 66.9% **Fig 2B**). These associations were significant when tested with an ordinal logistic regression model and persisted after correcting for potential confounders (p = 0.0002, **Table 4**). When exploring the proportion of patients with adequate LDL control across UACR categories or the proportion of patients with adequate control of any of the three drugable risk factors across eGFRcrea categories, we found no clear trend for LDL control across UACR categories or for control of any of the three examined risk factors across the eGFRcrea categories (**Fig 2C).** Combining eGFRcrea categories 4 and 5 (i.e. eGFRcrea 15–29 ml/min/1.73m^2^ and <15ml/min/1.73m^2^) did not substantially change the results (data not shown).

**Table 4:** Association of adequate blood pressure and HbA1c factor control with UACR categories in the 2892 analysed DIACORE patients.

	Beta (SE) for higher UACR category	P for association with higher UACR category
*Adequate blood pressure control*		
**Univariable**	-0.32 (0.04)	<0.0001
**Model 1**	-0.29 (0.05)	<0.0001
**Model 2**	-0.36 (0.05)	<0.0001
*Adequate HbA1c control*		
**Univariable**	-0.29 (0.05)	<0.0001
**Model 1**	-0.26 (0.05)	<0.0001
**Model 2**	-0.20 (0.05)	0.0002

Independent variable: adequate blood pressure control (RR<140 and <90 mmHg = 1, RR ≥140 or ≥90mmHg = 0) or adequate HbA1c control (HbA1c<7.5% = 1, HbA1c≥7.5% = 0). Dependent variable: albuminuria category (UACR<30mg/g: 0 [lower category]; UACR 30-300mg/g:1; UACR>300mg/g: 2 [higher category]) Shown are beta estimates, standard errors (SE) and p-values from the ordinal logistic regression models without (univariable) and with adjustment for potential confounders (model 1: model with additional adjustment for age, sex, diabetes duration; model 2: model 1 additionally adjusting for eGFR, BMI, waist-hip-ratio, smoking status, HbA1c/systolic and diastolic blood pressure).

The percentage refers to the patients within each CKD category shown in Fig 1. Field coloring indicates risk for progression of CKD according to the 2012 KDIGO guideline (green: low risk, yellow: moderately increased risk, orange: high risk, red: very high risk) [25]. (A) Distribution of the analysed 2892 DIACORE patients with the recommended target for blood pressure (<140/90mmHg) within each KDIGO 2012 CKD category. (B) Distribution of the analysed 2892 DIACORE patients with the recommended target for HbA1c (<58.0 mmol/mol; <7.5%)) within each KDIGO 2012 CKD category. (C) Distribution of the analysed 2892 DIACORE patients with the recommended target for LDL (<100mg/dl) within each KDIGO 2012 CKD category.

## Discussion

This analysis of the baseline visit of the DIACORE study provides important insights into an unselected outpatient DM2 collective: first, we observed the expected high prevalence of macro- and microvascular comorbidity. Second, blood pressure, HbA1c and lipid goals were attained in only 55.7%, 78.5% and 34.4% of patients respectively, despite a high number of prescribed drugs. This indicates a large potential for reducing macro- and microvascular risk in DM2 patients. Finally, adequate control of blood pressure and HbA1c control was significantly associated with decreased risk for higher categories of albuminuria levels.

This analysis of the DIACORE baseline study visit provides important insights into risk factor control and micro- and macrovascular disease burden in DM2 patients typically seen in an outpatient setting. Since we did not apply a certain level of cardiovascular risk or burden as an inclusion criterion, DIACORE extends information gained from large randomized controlled trials (RCT’s) performed in DM2 patients (e.g., ADVANCE, ACCORD, EMPA-REG, ALTITUDE, SUSTAIN-6, VADT), which were ascertained for having a particularly high cardiovascular risk profile [10–12, 15, 16, 21, 29–31]. Consistent with this, we observe that mean HbA1c in DIACORE is lower, macrovascular complications are less frequent and prevalence of DKD is lower than in most of the cited DM2 RCT’s. Baseline antihypertensive and glucose lowering therapy in DIACORE and current RCT’s is largely comparable, with 79.4%, 84.9–86.0% and 75.1% of patients under antihypertensive therapy and 64.2%, 59.7–60% and 60.2–61% under biguanide therapy in DIACORE, ACCORD and ADVANCE respectively. Only 1.5% of patients were under insulin treatment in the ADVANCE study versus 32.9% in the DIACORE and 34.1–35.7% in the ACCORD study, since insulin therapy was an exclusion criterion in ADVANCE. The use of statins differed most between the studies, with only 27.9% of patients in the ADVANCE study taking a statin versus 61.7–62.4% in ACCORD and 46.9% in DIACORE, with subsequent differences in LDL-control [10–12, 15].

Our study sample compares well to the German subgroup (n = 959) of the retrospective GUIDANCE study, a cross-sectional survey of DM2 patients recruited in primary and specialist sites in 8 European countries [32]. There was a comparable degree of medication prescription and risk factor control in DIACORE and the German GUIDANCE DM2 subgroup. However, patients had more macro- and microvascular complication in the GUIDANCE study: with 24.8% and 30.1% of patients having any macrovascular complication, and 27.5% and 37.4% of patients having any microvascular complication in DIACORE and GUIDANCE respectively. This difference in prevalence of microvascular events between DIACORE and GUIDANCE may be explained through the fact that GUIDANCE includes nonpalpable tibial or dorsal pulses as peripheral arterial disease and foot sensation abnormalities as well as blindness as microvascular events. Taken together, the good comparability of DIACORE with GUIDANCE underscores the value of DIACORE’s clinical data.

The lack of adequate risk factor control observed in a large proportion of DIACORE patients points to potential shortcomings in the care of patients with diabetes. Improvements may be achieved by e.g. more stringent disease management strategies or by novel pharmaceutical strategies. Specifically, recently published positive RCT’s of novel drug classes (SGLT2 inhibitors, GLP-1- receptor agonists) [33–35], not frequently used in DIACORE’s baseline visit, will likely change the pattern of drug use in patients with diabetes. Our ongoing longitudinal evaluations measuring risk factors at each 2 year follow-up visit will provide more details on the extent of risk factor control in this patient group in the future.

Strengths of our study include the broad scope of DM2 patients recruited into this baseline visit. Further strengths include the assessment of key laboratory measurements in a central facility and the medical validation of patient reported comorbidities at each study visit. However, some limitations warrant to be mentioned: first, though we were able to recruit a large proportion of patients with DM2 identified by the insurance companies’ records, the observed 16.9% response to the mailing by insurance companies and performance of examinations at the study site may have introduced bias through non-response of patients with limited mobility (e.g. patients not able to leave a nursing facility) or severe morbidity (e.g. patients in hospitals). Second, except for determination of CKD stage, our assessment of vascular complications is retrospective in this baseline data as presented here. However, by our stringent validation of self-report with medical records, there is a large credibility in reported proportions. Third, the presented data is cross-sectional and cannot yet provide insights into risk factors or biomarkers for incident complications of DM2, and associations cannot imply causality. However, by our cohort design with 2-year follow-up visits at our study centers, taking medical exams and blood repeatedly, we will be able to gain insights into incident diabetes-associated complications. Thus, we will be able to compare our incidence rates of diabetes complications with that of other prospective studies such as UKPDS [36]. Finally, since we bio-bank blood and urine samples gathered at baseline and at each 2 year follow-up visit, we will have a substantial resource for future research.

## Summary

DIACORE represents a large cohort of DM2 patients with a significant comorbidity, for which we present results from the baseline survey. Our findings suggest that there is a potential to further reduce macro- and microvascular burden through better risk factor control. Given the high comorbidity burden, further research on other disease mechanisms and risk factors is warranted. Due to its long-term follow-up as well as prospective data collection and bio-banking, we expect DIACORE to have an impact on future diagnostic and therapeutic strategies in DM2 patients.

## Supporting information

S1 Table Parameters determined in the DIACORE central laboratory panel at baseline. (DOCX)

S2 Table Source of information about DIACORE study. Shown are proportions of DIACORE study participants by source of information of the DIACORE.
(DOCX)

S3 Table Clinical characteristics of the 3000 DIACORE participants at the baseline visit. (DOCX)

S4 Table Comorbidities of the 3000 DIACORE participants at the baseline visit. (DOCX)

S5 Table Medication reported by 3000 DIACORE participants at the baseline visit. (DOCX)

S6 Table Clinical characteristics and risk factor values for the 2892 analyzed DIACORE participants by KDIGO categories of eGFRcrea and UACR. (DOCX)

S7 Table Risk factor control in the analyzed 2892 patients stratified by participation in a disease management program (DMP). (DOCX)

S1 Fig Recruitment into the DIACORE Study. (TIFF)

S2 Fig Distribution of male (S2A Fig) and female (S2B Fig) patients with nonmissing data in 9 key variables according to eGFR and albuminuria categories according the KDIGO 2012 CKD classification [25]. Field coloring indicates risk for progression of CKD according to the 2012 KDIGO guideline (green: low risk, yellow: moderately increased risk, orange: high risk, red: very high risk) [25].
(DOCX)
