# Supplementary material for: Poor risk factor control in outpatients with diabetes mellitus type 2 in Germany: The DIAbetes COhoRtE (DIACORE) study
Source: PLoS One. 2019 Mar 21;14(3):e0213157. doi: 10.1371/journal.pone.0213157 (PMC6428304; doi:10.1371/journal.pone.0213157)
Supplement: S1 Table — (DOCX) [file pone.0213157.s001.docx]

**Supplementary Table 1:** Parameters determined in the DIACORE central laboratory panel at baseline.

| **Parameter** | **Assay Name** | **Company** | **Analyzer used** |
| --- | --- | --- | --- |
| *Serum (Li-Heparin)* |  |  |  |
| Creatinine | CreaPlus, IDMS traceable | Roche Cobas | Roche Modular P Module |
| Cystatin C | Cystatin C Tina Quant | Roche Cobas | Roche Modular P Module |
| Urea | Urea/BUN kinetic UV test | Roche Cobas | Roche Modular P Module |
| Uric acid | UA plus | Roche Cobas | Roche Modular P Module |
| Sodium | ISE indirect Na, K, Cl for Gen.2 | Roche Cobas | Roche Modular P Module |
| Potassium | ISE indirect Na, K, Cl for Gen.2 | Roche Cobas | Roche Modular P Module |
| Calcium | Ca | Roche Cobas | Roche Modular P Module |
| Phophate | PHOS anorganic phosphate | Roche Cobas | Roche Modular P Module |
| Albumin | Albumin Tina Quant | Roche Cobas | Roche Modular P Module |
| hsCRP | CRPHS Tina Quant | Roche Cobas | Roche Modular P Module |
| Cholesterol | CHOL CHOD-PAP | Roche Cobas | Roche Modular P Module |
| Triglyceride | TG Triglyceride GPO-PAP | Roche Cobas | Roche Modular P Module |
| LDL | LDL-C plus 2nd generation | Roche Cobas | Roche Modular P Module |
| HDL | HDL-C plus 3rd generation | Roche Cobas | Roche Modular P Module |
| Insulin | Insulin | Roche Cobas | Roche Modular E170 |
| *Plasma (Na-F)* |  |  |  |
| Glucose | GLU Gluco-quant glucose/HK | Roche Cobas | Roche Modular P Module |
| *Whole Blood* |  |  |  |
| HbA1c | Hemoglobin A1c Gen.2 Tina Quant for whole blood | Roche Cobas | Roche Integra 800 |
| Whole blood count | NA | NA | Siemens ADVIA 2120i |
| *Urine* |  |  |  |
| Creatinine | CreaPlus, IDMS traceable | Roche Cobas | Roche Modular P Module |
| Albumin | Albumin Tina Quant | Roche Cobas | Roche Modular P Module |
|  |  |  |  |
|  |  |  |  |
