# Supplementary material for: Poor risk factor control in outpatients with diabetes mellitus type 2 in Germany: The DIAbetes COhoRtE (DIACORE) study
Source: PLoS One. 2019 Mar 21;14(3):e0213157. doi: 10.1371/journal.pone.0213157 (PMC6428304; doi:10.1371/journal.pone.0213157)
Supplement: S2 Table — Shown are proportions of DIACORE study participants by source of information of the DIACORE. (DOCX) [file pone.0213157.s002.docx]

**Supplementary Table 2: Source of information about DIACORE study.** Shown are proportions of DIACORE study participants by source of information of the DIACORE.

| **Mode of information** | **% (n)** |
| --- | --- |
| Invitation by insurance company | 34.3% (1030) |
| Press article | 26.3% (790) |
| Referral by diabetologist | 8.6% (258) |
| Referral by general practitioners | 1.7% (52) |
| Invitation by the University Hospital Regensburg | 7.6% (228) |
| Recruitment in diabetology office in Speyer | 17.05% (509) |
| Other means (radio interview, information from other participants, etc.) | 4.5% (133) |
| Total | 3000 |
