# Supplementary material for: Poor risk factor control in outpatients with diabetes mellitus type 2 in Germany: The DIAbetes COhoRtE (DIACORE) study
Source: PLoS One. 2019 Mar 21;14(3):e0213157. doi: 10.1371/journal.pone.0213157 (PMC6428304; doi:10.1371/journal.pone.0213157)
Supplement: S3 Table — (DOCX) [file pone.0213157.s003.docx]

**Supplementary Table 3:**  Clinical characteristics of the 3000 DIACORE participants at the baseline visit.

|  | **Total** | **Men** | **Women** |
| --- | --- | --- | --- |
| N | 3000 (100%) | 1801 (60.0%) | 1199 (40.0%) |
| Age, years | 65.2±9.4 | 65.5±9.1 | 64.9±9.7 |
| Diabetes duration, years^#,&^ | 8.4 (4.1-14.7) | 9.1 (4.6-15.3) | 7.6 (3.6-12.9) |
| Disease management program, n (%) | 2307 (76.9%) | 1339 (74.3%) | 968 (80.7%) |
| **Cardiovascular risk factors** |  |  |  |
| HbA1c** , mmol/mol^#,§^ | 49 (44-57) | 50 (45-58) | 49 (44-55-8) |
| HbA1c** , % ^#,§§^ | 6.6 (6.2-7.4) | 6.7 (6.3-7.5) | 6.6 (6.2-7.3) |
| HbA1c** < 7.5 %, n (%) | 2345 (78.4%) | 1383 (77.0%) | 962 (80.4%) |
| Glucose in patients fasting >12 h^#,^***, mg/dl | 122 (103.1-147.4) | 126 (106.7-150) | 116 (98.6-140) |
| Glucose in nonfasting patients, mg/dl^#^ | 115.9 (94.9-154.8) | 123.6 (98.1-165.7) | 107 (90.5-137) |
| Systolic blood pressure, mmHg**** | 139.0±18.1 | 139.4±17.7 | 138.5±18.7 |
| Diastolic blood pressure, mmHg**** | 76.5±10.5 | 77.9±10.4 | 74.3±10.4 |
| Blood pressure <140/90 mmHg, n (%) | 1665 (55.5%) | 972 (54.0%) | 693 (57.8%) |
| BMI^$^, kg/m² | 31.4±5.7 | 31.0±5.3 | 32.0±6.2 |
| WHR^$^ | 0.96±0.08 | 1.00±0.06 | 0.89±0.07 |
| HDL**, mg/dl | 52.9±15.3 | 49.3±13.6 | 58.4±16.0 |
| LDL**, mg/dl | 118.0±37.0 | 112.5±35.7 | 126.4±37.2 |
| LDL** < 100 mg/dl, n (%) | 1009 (33.7%) | 716 (39.9%) | 293 (24.5%) |
| Never-smokers, n (%) | 1263 (42.3%) | 521 (29.0%) | 742 (62.0%) |
| Current smokers, n (%) | 380 (12.7%) | 249 (13.9%) | 131 (10.9%) |
| Former smokers, n (%) | 1345 (45.0%) | 1021 (57.0%) | 324 (27.0%) |

Data are presented as mean (SD) or % (n) if not indicated otherwise.

#: median (IQR)

**: Serum and whole blood samples were available in 2993 (99.8%) patients for measurement of LDL and HDL. Whole blood was available in 2992 (99.7%) patients for measurement of HbA1c.

*** 1670 patients (55.7%) were fasting > 12h.

****: Blood pressure was available in 2998 (99.9%) patients.

$: BMI available in 2985 (99.5%), WHR in 2943 (98.1%).

& mean (yrs): 10.3±8.4 (overall), 11.0±8.8 (men), 9.3±7.6 (women).

§ mean (mmol/mol)52.2±12.3 (overall), 52.7±12.6 (men), 51.5±11.8 (women).

§§ mean (%) 6.9±1.1 (overall), 7.0±1.1 (men), 6.9±1.1 (women).
