# Supplementary material for: Poor risk factor control in outpatients with diabetes mellitus type 2 in Germany: The DIAbetes COhoRtE (DIACORE) study
Source: PLoS One. 2019 Mar 21;14(3):e0213157. doi: 10.1371/journal.pone.0213157 (PMC6428304; doi:10.1371/journal.pone.0213157)
Supplement: S4 Table — (DOCX) [file pone.0213157.s004.docx]

**Supplementary Table 4:** Comorbidities of the 3000 DIACORE participants at the baseline visit.

|  | **Total**  **n=3000** | **Men**  **n=1801** | **Women**  **n=1199** |
| --- | --- | --- | --- |
|  |  |  |  |
| **Kidney function and albuminuria*** |  |  |  |
| Serum creatinine, mg/dl ^#,^§ | 0.89 (0.75-1.07) | 0.96 (0.85-1.15) | 0.76 (0.66-0.9) |
| Serum cystatin C, mg/dl ^#,^$ | 0.98 (0.86-1.16) | 1.0 (0.87-1.17) | 0.97 (0.85-1.15) |
| eGFRcrea CKD-EPI, ml/min/1.73m² ^#,&^ | 82.2 (65.9-93.4) | 81.3 (65.7-92.4) | 83.1 (66.1-94.8) |
| eGFRcys CKD-EPI, ml/min/1.73m² ^#,^** | 75.6 (59.4-91.1) | 76.3 (60.4-92.3) | 74.2 (58.1-88.8) |
| UACR, mg/g ^#^ | 10.1 (4.8-31.1) | 11.4 (4.7-42.7) | 9.0 (5.0-21.2) |
|  |  |  |  |
| **Microvascular comorbidities** |  |  |  |
| Diabetic retinopathy |  |  |  |
| Previous retinal laser therapy | 114 (3.8%) | 77 (4.3%) | 37 (3.1%) |
| Diabetes associated kidney disease |  |  |  |
| eGFRcrea <60 ml/min/1.73m² or UACR≥30mg/g, n (%) | 1099 (37.5%) | 729 (41.4%) | 370 (31.5%) |
| CKD stage 3 or higher (eGFRcrea<60 ml/min/1.73m²) | 19.0% (568) | 19.4% (349) | 18.2% (219) |
| UACR 30-300 mg/g | 21.3% (619) | 25.1% (440) | 15.4% (179) |
| UACR>300mg/g | 4.5% (130) | 5.7% (100) | 2.6% (30) |
|  |  |  |  |
| **Macro-vascular complications** |  |  |  |
| Myocardial infarction | 255 (8.5%) | 216 (12.0%) | 39 (3.3%) |
| Operative myocardial revascularisation | 197 (6.6%) | 171 (9.5%) | 26 (2.2%) |
| Percutaneous coronary intervention | 398 (13.3%) | 329 (18.3%) | 69 (5.8%) |
| Stroke | 201 (6.7%) | 137 (7.6%) | 64 (5.3%) |
| Operative or percutaneous carotid intervention | 76 (2.5%) | 59 (3.3%) | 17 (1.4%) |
| Revascularization lower extremities | 51 (1.7%) | 44 (2.4%) | 7 (0.6%) |
| Amputation | 61 (2.0%) | 53 (2.9%) | 8 (0.7%) |
|  |  |  |  |

Data are presented as mean (SD) or % (n) if not indicated otherwise.

*: Serum and whole blood samples were available in 2993 (99.8%) patients for measurement of serum creatinine and cystatin C. Urine samples were available in 2913 (97.1%) of patients for measurement of urinary albumin and creatinine.

#: median (interquartile range).

§ mean (mg/dl): 0.96±0.4 (overall), 1.05±0.4 (men), 0.8±0.3 (women).

$ mean (mg/dl): 1.1±0.4 (overall), 1.1±0.4 (men), 1.1±0.4 (women).

& mean (ml/min/1.73m^2^): 78.5±20.4 (overall), 77.9±20.3 (men), 79.4±20.6 (women).

** mean (ml/min/1.73m^2^): 74.6±22.5 (overall), 75.6±23.1 (men), 73.2±21.6 (women).
