# Supplementary material for: Poor risk factor control in outpatients with diabetes mellitus type 2 in Germany: The DIAbetes COhoRtE (DIACORE) study
Source: PLoS One. 2019 Mar 21;14(3):e0213157. doi: 10.1371/journal.pone.0213157 (PMC6428304; doi:10.1371/journal.pone.0213157)
Supplement: S5 Table — (DOCX) [file pone.0213157.s005.docx]

**Supplementary Table 5** Medication reported by 3000 DIACORE participants at the baseline visit.

|  | **Total** | Male | Female |
| --- | --- | --- | --- |
| **Glucose lowering medication n, (%)** | **2628 (87.6%)** | 1584 (88.0%) | 1044 (87.0%) |
| Injectable, n (%) | **1039 (34.6%)** | 667 (37.0%) | 372 (31.0%) |
| Insulin, n (%) | **988 (32.9%)** | 639 (35.5%) | 349 (29.1%) |
| GLP-1 receptor agonists, n (%) | **63 (2.1%)** | 36 (2.0%) | 27 (2.3%) |
| Oral Antidiabetic medication, n (%) | **2238 (74.6%)** | 1330 (73.8%) | 908 (75.7%) |
| Biguanides, n (%) | **1927 (64.2%)** | 1145 (63.6%) | 782 (65.2%) |
| Sulfonylureas, n (%) | **567 (18.9%)** | 365 (20.3%) | 202 (16.9%) |
| DPP4-inhibitor, n (%) | **578 (19.3%)** | 362 (20.1%) | 216 (18.0%) |
| Glinides, n (%) | **121 (4.0%)** | 78 (4.3%) | 43 (3.9%) |
| Alphaglukosidase-Inhibitors, n (%) | **50 (1.7%)** | 33 (1.8%) | 17 (1.4%) |
| SGLT2-Inhibitors | **12 (0.4%)** | 8 (0.4%) | 4 (0.3%) |
| **Antihypertensive medication, n (%)** | **2420 (80.7%)** | 1475 (81.9%) | 945 (78.8%) |
| RAAS-Inhibitor, n (%) | **2009 (67.0%)** | 1262 (70.7%) | 747 (62.3%) |
| ACE-Inhibitor, n (%) | **1279 (42.6%)** | 855 (47.5%) | 424 (35.4%) |
| Angiotensin receptor blocker, n (%) | **754 (25.1%)** | 422 (23.4%) | 323 (27.7%) |
| Renin Inhibitor, n (%) | **34 (1.1%)** | 22 (1.0%) | 12 (1.0%) |
| Calcium channel blocker, n (%) | **835 (27.8%)** | 526 (29.2%) | 309 (25.8%) |
| Beta-Blocker, n (%) | **1435 (47.8%)** | 887 (49.3%) | 548 (45.7%) |
| Diuretics, n (%) | **1248 (41.6%)** | 741 (41.1%) | 507 (42.3%) |
| **Lipid lowering medication, n (%)** | **1462 (48.7%)** | 933 (51.8%) | 529 (44.1%) |
| Statin, n (%) | **1406 (46.9%)** | 894 (49.6%) | 512 (42.7%) |
| Fibrate, n (%) | **71 (2.4%)** | 51 (2.8%) | 20 (1.7%) |
