# Supplementary material for: Poor risk factor control in outpatients with diabetes mellitus type 2 in Germany: The DIAbetes COhoRtE (DIACORE) study
Source: PLoS One. 2019 Mar 21;14(3):e0213157. doi: 10.1371/journal.pone.0213157 (PMC6428304; doi:10.1371/journal.pone.0213157)
Supplement: S6 Table — (DOCX) [file pone.0213157.s006.docx]

**Supplementary Table 6*:*** Clinical characteristics and risk factor values for the 2892 analyzed DIACORE participants by KDIGO categories of eGFRcrea and UACR.

|  | **eGFR** | | | | | **UACR** | | |
| --- | --- | --- | --- | --- | --- | --- | --- | --- |
|  | **G1** | **G2** | **G3** | **G4** | **G5** | **A1** | **A2** | **A3** |
| N | 979 | 1370 | 479 | 60 | 4 | 2149 | 613 | 130 |
| Age, years | 58.5 (58.0-59.1) | 67.6 (67.2-67.9) | 71.7 (71.0-72.3) | 71.3 (68.7-73.9) | 74.8 (64.7-84.9) | 64.8 (64.4-65.2) | 66.7 (65.9-67.4) | 66.4 (64.7-68.1) |
| Diabetes duration, years | 8.1 (7.7-8.5) | 10.5 (10.0-10.9) | 13.6 (12.8-14.5) | 15.5 (12.8-18.2) | 19.6 (-6.3-45.6) | 9.6 (9.2-9.9) | 12.0 (11.2-12.7) | 14.7 (13.1-16.3) |
| Disease management program, n (%) | 765 (78.1%) | 1023 (75.0%) | 383 (80.0%) | 50 (83.3%) | 4 (100%) | 1661 (77.3%) | 458 (74.9%) | 105 (80.8%) |
| **Cardiovascular risk factors** |  |  |  |  |  |  |  |  |
| HbA1c** , % | 7.0 (6.96-7.1) | 6.8 (6.78-6.9) | 6.9 (6.8-7.0) | 7.5 (7.0-8.0) | 6.5 (5.5-7.5) | 6.9 (6.8-6.9) | 7.1 (7.0-7.2) | 7.3 (7.0-7.5) |
| HbA1c** < 7.5 %, n (%) | 726 (74.2%) | 1127 (82.3%) | 374 (78.1%) | 38 (63.3%) | 4 (100%) | 1744 (81.2%) | 438 (71.5%) | 87 (66.9%) |
| Systolic blood pressure, mmHg**** | 137.4 (136.4-138.5) | 140.2 (139.3-141.2) | 138.8 (137.0-140.5) | 136.7 (130.4-142.9) | 140.4 (107.0-173.8) | 136.9 (136.2-137.6) | 143.9 (142.3-145.5) | 149.7 (146.2-153.2) |
| Diastolic blood pressure, mmHg**** | 79.0 (78.4-79.7) | 76.4 (75.8-76.9) | 72.6 (71.7-73.5) | 69.6 (66.9-72.3) | 73.4 (58.9-87.9) | 76.1 (75.6-76.5) | 77.8 (76.9-78-7) | 77.9 (76.1-79.7) |
| Blood pressure <140/90 mmHg, n (%) | 569 (58.1%) | 722 (52.7%) | 279 (58.2%) | 38 (63.3%) | 2 (50%) | 1283 (59.7%) | 281 (45.8%) | 46 (35.4%) |
| BMI^$^, kg/m² | 31.5 (31.1-31.9) | 31.0 (30.7-31.3) | 32.2 (31.7-32.7) | 32.7 (31.1-34.2) | 32.0 (17.9-46.1) | 31.1 (30.9-31.3) | 32.2 (31.7-32.7) | 32.8 (31.7-33.9) |
| WHR^$^ | 0.96 (0.95-0.96) | 0.96 (0.96-0.96) | 0.97 (0.97-0.98) | 0.97 (0.95-1.0) | 1.0 (0.97-1.04) | 1.0 (0.95-0.96) | 0.98 (0.98-0.99) | 1.0 (0.98-1.0) |
| HDL**, mg/dl | 53.3 (52.3-54.2) | 54.2 (53.5-55.0) | 49.7 (48.4-50.9) | 45.7 (42.3-49.0) | 40.8 (29.7-51.8) | 53.7 (53.1-54.4) | 50.6 (49.4-51.8) | 50.4 (47.6-53.1) |
| LDL**, mg/dl | 120.0 (117.6-122.1) | 118.8  (116.8-120.7) | 112.8 (109.5-116.1) | 107.5 (95.4-119.6) | 123.2 (39.9-206.6) | 119.7 118.2-121.3) | 112.0 (109.1-114.8) | 116.5 (108.5-124.4) |
| LDL** < 100 mg/dl, n (%) | 280 (28,6%) | 463 (33,8%) | 206 (43.0%) | 27 (45.0%) | 2 (50%) | 685 (31.9%) | 248 (40.5%) | 45 (34.6%) |
| Never-smokers, n (%) | 376 (38.6%) | 612 (45.0%) | 208 (43.5%) | 24 (40.0%) | 1 (25.0%) | 958 (44.7%) | 227 (37.2%) | 36 (28.1%) |
| Current smokers, n (%) | 183 (18.8%) | 129 (9.5%) | 39 (8.2%) | 6 (10.0%) | 1 (25.0%) | 258 (12.0%) | 77 (12.6%) | 23 (18.0%) |
| Former smokers, n (%) | 416 (42.7%) | 622 (45.6%) | 231 (48.3%) | 30 (50.0%) | 2 (50.0%) | 926 (43.2%) | 50.2%) | 69 (53.9%) |

Data are presented as mean (95% confidence interval) or n(%).
