# Supplementary material for: Poor risk factor control in outpatients with diabetes mellitus type 2 in Germany: The DIAbetes COhoRtE (DIACORE) study
Source: PLoS One. 2019 Mar 21;14(3):e0213157. doi: 10.1371/journal.pone.0213157 (PMC6428304; doi:10.1371/journal.pone.0213157)
Supplement: S7 Table — (DOCX) [file pone.0213157.s007.docx]

**Supplementary Table 7:** Risk factor control in the analyzed 2892 patients stratified by participation in a disease management program (DMP).

|  | **Total** | **DMP** | **no DMP** |  |
| --- | --- | --- | --- | --- |
| n * | 2892 (100%) | 2225 (76.9%) | 611 (21.1%) |  |
| Blood pressure <140/90 mmHg, n (%) | 1610 (55.6%) | 1242 (55.8 %) | 337 (55.1 %) | p=0.95 |
| LDL < 100 mg/dl, n (%) | 978 (33.8%) | 756 (34.0 %) | 206 (33.7 %) | p=0.69 |
| HbA1c < 58.0mmol/mol , n(%) | 2269 (78.5%) | 1740 (78.2 %) | 483 (79.1%) | p=0.71 |

***** For 56 of 2892 patients, data on participation in a DMP was not availble.
