# Supplementary figures and images for: Poor risk factor control in outpatients with diabetes mellitus type 2 in Germany: The DIAbetes COhoRtE (DIACORE) study
Source: PLoS One. 2019 Mar 21;14(3):e0213157. doi: 10.1371/journal.pone.0213157 (PMC6428304; doi:10.1371/journal.pone.0213157)

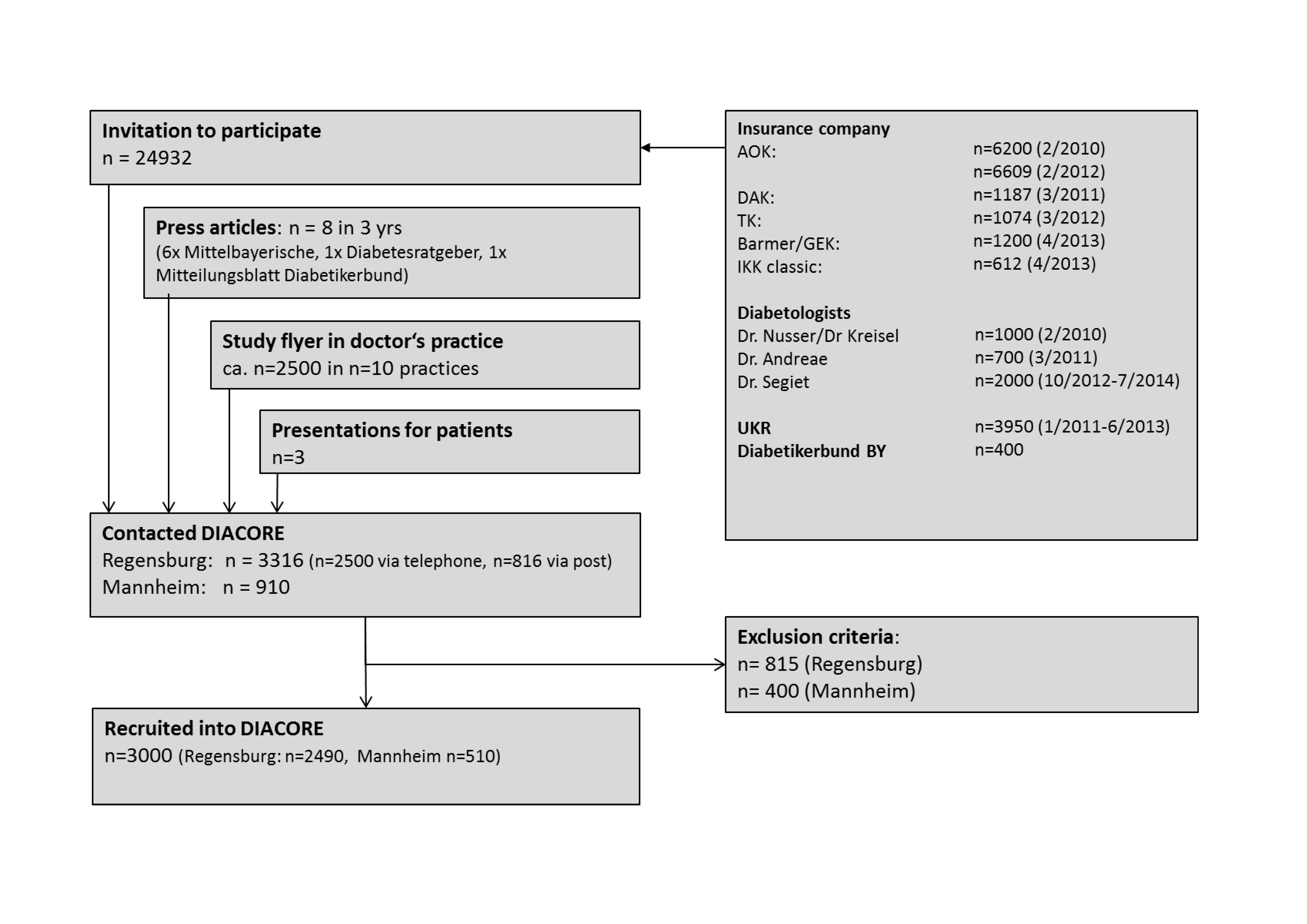

Supplement: S1 Fig — (TIFF) [file pone.0213157.s008.tiff]

S2a Fig


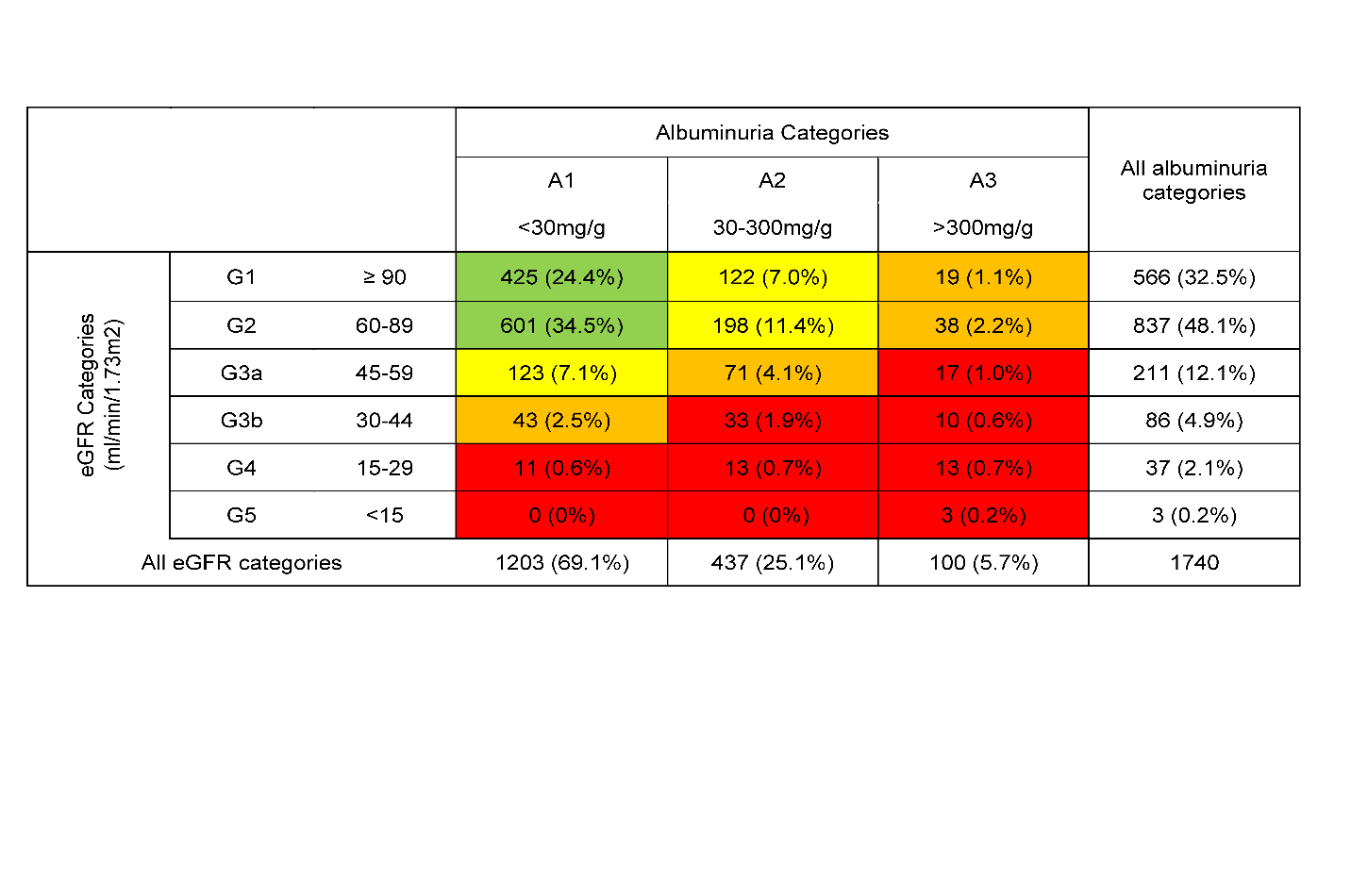
 S2b Fig


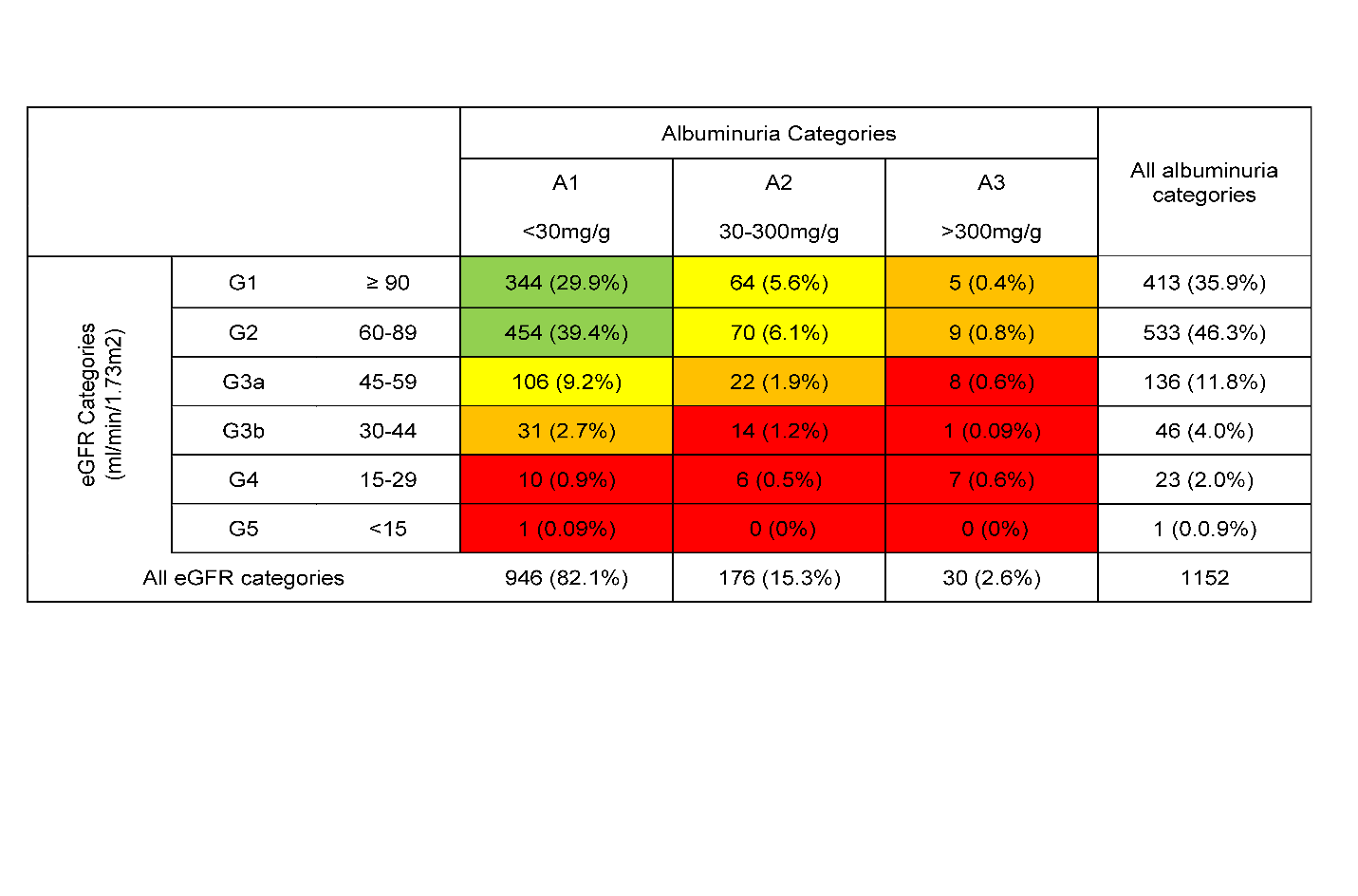

Supplement: S2 Fig — Field coloring indicates risk for progression of CKD according to the 2012 KDIGO guideline (green: low risk, yellow: moderately increased risk, orange: high risk, red: very high risk) [25]. (DOCX) [file pone.0213157.s009.docx]
